# Treating Protein Misfolding Diseases: Therapeutic Successes Against Systemic Amyloidoses

**DOI:** 10.3389/fphar.2020.01024

**Published:** 2020-07-10

**Authors:** Alice Nevone, Giampaolo Merlini, Mario Nuvolone

**Affiliations:** ^1^ Amyloidosis Research and Treatment Center, Foundation IRCCS Policlinico San Matteo, Pavia, Italy; ^2^ Department of Molecular Medicine, University of Pavia, Pavia, Italy

**Keywords:** protein misfolding (conformational) diseases, AL amyloidosis, ATTR amyloidosis, AA amyloidosis, amyloidosis, early intervention

## Abstract

Misfolding and extracellular deposition of proteins is the hallmark of a heterogeneous group of conditions collectively termed protein misfolding and deposition diseases or amyloidoses. These include both localized (e.g. Alzheimer’s disease, prion diseases, type 2 diabetes mellitus) and systemic amyloidoses. Historically regarded as a group of maladies with limited, even inexistent, therapeutic options, some forms of systemic amyloidoses have recently witnessed a series of unparalleled therapeutic successes, positively impacting on their natural history and sometimes even on their incidence. In this review article we will revisit the most relevant of these accomplishments. Collectively, current evidence converges towards a crucial role of an early and conspicuous reduction or stabilization of the amyloid-forming protein in its native conformation. Such an approach can reduce disease incidence in at risk individuals, limit organ function deterioration, promote organ function recovery, improve quality of life and extend survival in diseased subjects. Therapeutic success achieved in these forms of systemic amyloidoses may guide the research on other protein misfolding and deposition diseases for which effective etiologic therapeutic options are still absent.

## Protein Misfolding Diseases

Protein misfolding and deposition diseases arise when one of an ever growing list of proteins (the amyloid-forming protein in its native conformation, also referred to as the amyloidogenic precursor) acquires an alternative folding state (the misfolded state), starts to aggregate and to form oligomers, then protofibrils and finally fibrillar structures (termed amyloid fibrils) which eventually deposit within tissues (forming the amyloid deposits) (Benson et al., 2018a). This process can occur at the site of protein production, leading to localized amyloidoses, as it is the case of Alzheimer’s disease and the majority of prion diseases affecting the central nervous system (CNS) and type 2 diabetes mellitus affecting the islets of Langerhans within the pancreas (Aguzzi et al., 2013; Westermark and Westermark, 2013; Selkoe and Hardy, 2016). Conversely, amyloid deposition can affect multiple body sites (typically sparing the central nervous system parenchyma) when the amyloid-forming protein is a circulating protein, as in the case of systemic amyloidoses (Nuvolone and Merlini, 2017b) (**Table 1**). The prion protein can form amyloid deposits outside of the CNS in the presence of selected mutations of the *PRNP* gene, leading to PrP systemic amyloidosis (Mead et al., 2013; Matsuzono et al., 2016; Capellari et al., 2018), or in the context of variant Creutzfeldt-Jakob disease (Will et al., 1996; Bruce et al., 2001). Rarely, iatrogenic amyloid deposits can be formed at the site of drug injection, as reported for insulin and enfuvirtide (Storkel et al., 1983; Morilla et al., 2009).

**Table 1:** Localized versus systemic forms of amyloidosis.

	Localized amyloidoses				Systemic amyloidoses		
	Alzheimer’s disease	Prion diseases	Type 2 diabetes	Localized AL amyloidosis	Systemic AL amyloidosis	AA amyloidosis	ATTR amyloidosis
Source	CNS	CNS	Pancreas	Extra-medullary plasma cell clone	BM plasma cell clone	Liver	Liver Choroid plexus Retina
Amyloid-forming protein	Aβ	PrP	IAPP	Locally secreted monoclonal LC	Circulating monoclonal LC	SAA	TTR
Site of amyloid deposit	CNS	CNS#	Pancreas	One site: • Urogenital tract • Larynx • Skin • Lung • GI tract	• Heart • Kidney • Liver • GI tract • Peripheral nerves • Soft tissue	• Kidney • Liver • GI tract • Spleen • Thyroid	• Heart • Peripheral nerves • Carpal tunnel • Meninges • Eye

^#^Systemic amyloid deposits can be observed in some forms of prion diseases, including variant Creutzfeldt-Jacob disease and in PrP systemic amyloidosis. Aβ, amyloid β; BM, bone marrow; CNS, central nervous system; GI tract, gastrointestinal tract; IAPP, islet amyloid polypeptide; LC, immunoglobulin light chain; PrP, prion protein; SAA, serum amyloid A; TTR, transthyretin.

The complex process of protein misfolding, aggregation, amyloid oligomer and fibril formation and deposition can lead to cytotoxicity, subversion of tissue architecture, progressive organ damage, and eventually death (Merlini and Bellotti, 2003). Notwithstanding, amyloid itself may not be necessarily toxic, as witnessed by the existence of so-called functional amyloids within the body (Chiti and Dobson, 2006).

Sometimes amyloidogenesis occurs when a protein concentration is persistently elevated, like for the acute phase reactant serum amyloid A (SAA) in systemic amyloidosis associated to chronic inflammation (AA amyloidosis) or wild-type β_2_-microglobulin in systemic amyloidosis associated to dialysis (Westermark et al., 2015; Kaneko and Yamagata, 2018). In other cases, amyloidosis can be caused by genetic mutations which destabilize an otherwise non-amyloidogenic or only mildly amyloidogenic protein, as in familial prion diseases and hereditary systemic amyloidoses (including ATTRv amyloidosis associated to variant transthyretin, TTR) (Connors et al., 2003; Rowczenio et al., 2019), or by mutations which increase the synthesis or the proteolytic release of the amyloid-forming protein, as in the case of AA amyloidosis associated to hereditary autoinflammatory syndromes or familial Alzheimer’s disease, respectively (De Strooper et al., 2012; Obici and Merlini, 2012). In systemic amyloidosis associated to immunoglobulin light chains (AL amyloidosis), amyloidogenesis is the result of the presence of elevated concentrations of a patient’s unique, unstable light chain produced by an underlying B-cell clonal disorder (Merlini et al., 2018).

In some other instances, amyloid formation occurs in the absence of other apparent predisposing conditions, as a process associated to aging, presumably due to the intrinsic amyloidogenicity of a specific protein, as in the case of sporadic forms of Alzheimer’s and prion diseases, as well as in the case of systemic amyloidosis associated to wild-type TTR (ATTRwt amyloidosis) (Prusiner, 2001; Gertz et al., 2015; Long and Holtzman, 2019). Historically regarded as a group of conditions with limited, even inexistent, therapeutic options, some forms of systemic amyloidoses have recently witnessed a series of unparalleled therapeutic successes, positively impacting on their natural history and sometimes even on their incidence. Here we will review the most relevant of these accomplishments in order to draw some lessons which may guide research on other protein misfolding diseases for which effective etiologic therapeutic options are still absent.

## Elimination or Reduction of the Amyloid-Forming Protein Translates into Clinical Benefit

Substantial therapeutic success has been achieved by interventions aimed at eliminating or substantially reducing the supply of the amyloid-forming protein, thus interfering with the process of misfolding, oligomerization and amyloid formation (Nuvolone and Merlini, 2017b) (**Table 2**).

**Table 2:** Therapeutic interventions against the most common forms of systemic amyloidosis.

Strategy	AL amyloidosis	AA amyloidosis	ATTR amyloidosis
Eliminate or reduce the amyloid-forming protein	**Anti-PC (or B cells) drugs:** • Chemotherapy/ASCT • Immunomodulatory drugs • Steroids • Monoclonal antibodies	**Treatment of underlying flogosis:** • Steroids • DMARDs • Monoclonal antibodies • Antibiotics • Surgery • …	**TTR directed interventions:** • Liver transplantation (hATTR)° • TTR tetramer stabilizers @ • *TTR* silencing agents #
Monitor the concentration of the amyloid-forming protein	**Amyloid-forming protein:** monoclonal LC**Goal:** disappearance **Monitoring:** s+uEP+IFIX serum free LC levels	**Amyloid-forming protein:** SAA **Goal:** SAA normalization **Monitoring:** serum SAA levels	**Amyloid-forming protein:** TTR **Goal:** reduced mutant TTR° tetramer stabilization @ reduced TTR levels # **Monitoring:** tetramer stability/dissociation @ or serum TTR levels #@
Early diagnosis	**At risk individuals:** MGUS, MM, other PC dyscrasia **Proposed screening:** • Proteinuria • NT-proBNP • ALP	**At risk individuals:** Chronic inflammation, morbid obesity (?) **Proposed screening:** • Proteinuria	**At risk individuals:** pre-symptomatic mutation carriers (hATTR) **Proposed screening** **(based on *TTR* mutation):** • NT-proBNP • Nerve MRI

ALP, alkaline phosphatase; ASCT, autologous stem cell transplantation; BM, bone marrow; DMARDs, disease-modifying anti-rheumatic drugs; hATTR, hereditary ATTR amyloidosis; LC, immunoglobulin light chains; MGUS, monoclonal gammopathy of undetermined significance; MM, multiple myeloma; MRD, minimal residual disease; MRI, magnetic resonance imaging; NT-proBNP, amino-terminal pro brain natriuretic peptide; PC, plasma cell; SAA, serum amyloid A; s&uEP+IFIX, serum and urine electrophoresis with immunofixation; TTR, transthyretin. °, @ and # denote different therapeutic strategies in ATTR amyloidosis, with different goals, and whose efficacy can be monitored by different modalities.

**Figure 1:**
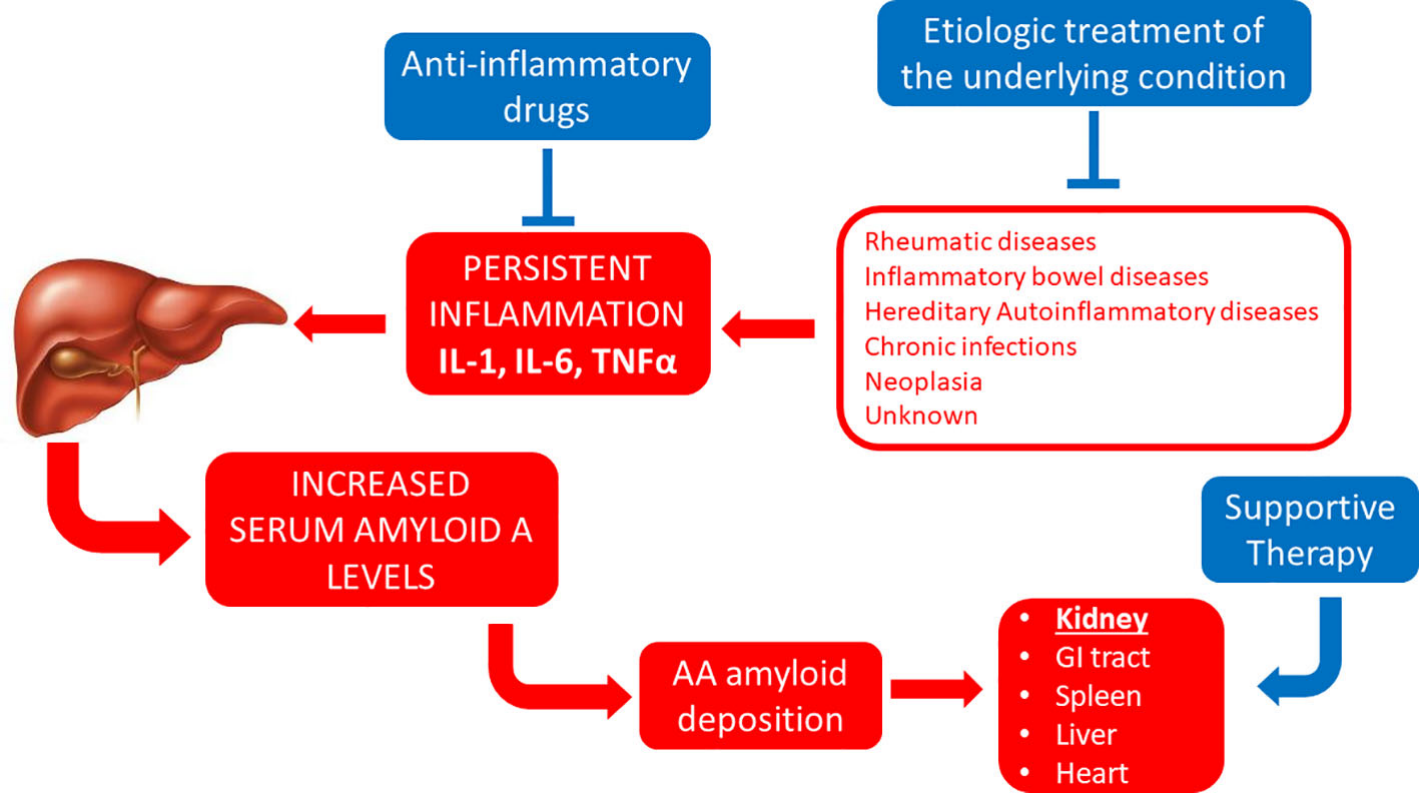
Established therapeutic interventions against AA amyloidosis. Red blocks indicate steps in the pathogenesis of AA amyloidosis; blue blocks indicate established therapeutic interventions against AA amyloidosis. IL-1, interleukin 1; IL-6, interleukin 6; GI tract, gastro-intestinal tract; TNFα, tumor necrosis factor α.

The first known examples of successful therapeutic approaches against systemic amyloidoses have been described in the context of AA amyloidosis (**Figure 1**). This is a form of systemic amyloidosis complicating longstanding chronic inflammation and most commonly arises in association with chronic arthritides, hereditary autoinflammatory syndromes, chronic inflammatory bowel diseases or chronic infections (**Table 3**). Amyloid deposits derive from the proteolytic processing of the liver-derived acute phase reactant SAA and almost invariably affect kidneys.

**Table 3:** Chronic inflammatory conditions underlying AA amyloidosis.

Class of underlying disorder	
Chronic infections	Tuberculosis
	Leprosy
	Osteomyelitis
	Whipple disease
	Chronic infections as a consequence of:
	Cystic fibrosis
	Bronchiectasis
	Paraplegia
	Injection-drug abuse
	Epidermolysis bullosa
	Immunodeficiencies
	Others
Rheumatologic diseases	Ankylosing spondylitis
	Juvenile idiopathic arthritis
	Psoriatic arthropathy Rheumatoid arthritis
	Systemic vasculitis
	Others
Inflammatory bowel diseases	Crohn’s disease Ulcerative colitis
Hereditary Auto-inflammatory Disorders	Familial Mediterranean fever (FMF) Mevalonate kinase deficiency (MKD)
	Cryopyrin-associated periodic fever syndrome (CAPS)
	Tumor necrosis factor receptor associated periodic syndrome (TRAPS)
	Others
Neoplasia	Castelman’s disease
	Lymphoma
	Sarcoma
	Adenocarcinoma
	Mesothelioma
	Others
Unknown	Morbid obesity (?)

In 1928, Henning Waldenström reported the rapid resolution of hepatomegaly and liver amyloid deposits after successful surgical treatment of “lymphoid TBC fistulae” in a child presumably affected by AA amyloidosis (Waldenstrom, 1928). Similar successful regression of AA amyloid deposits has been described after surgical excision of localized Castleman’s disease leading to the resolution of the associated chronic inflammation (Perfetti et al., 1994; Lachmann et al., 2002; Mandreoli et al., 2002; Verbrugghe et al., 2005; Androulaki et al., 2007).

Studies of experimental murine AA amyloidosis show that AA amyloid can be indeed spontaneously cleared upon cessation of inflammation through redundant innate immune mechanisms (Nystrom and Westermark, 2012; Lundmark et al., 2013; Sponarova et al., 2013). They also demonstrate that, after apparent resolution of AA amyloid deposits, a relapse of inflammation can lead to a dramatic, rapid exacerbation of amyloid deposits, presumably due to residual AA amyloid fibrils resisting clearing mechanisms and serving as nucleating seeds, further stressing the importance of maintaining reduced SAA levels throughout the disease (Hawkins and Pepys, 1990; Simons et al., 2013).

To which extent SAA levels influence the natural history of AA amyloidosis has been substantiated by work of Lachmann and collaborators based on the largest series of patients with AA amyloidosis reported so far. This study shows that amyloid burden, renal outcome and overall survival all correlate with SAA levels in the course of the disease (Lachmann et al., 2007). In this series, amyloid regression was seen in 60% of patients in which therapeutic interventions against the underlying cause of systemic inflammation succeeded at lowering SAA levels below the threshold of 10 mg/L (Lachmann et al., 2007). This should be therefore regarded as the therapeutic goal when treating these patients.

For a subset of patients with AA amyloidosis, which has proportionally increased in more recent case series, the cause of the underlying chronic inflammatory status cannot be identified despite an intensive diagnostic workout (Lane et al., 2017a). Recent work has shown that some of these patients may benefit from empirical treatment with interleukin-1 inhibition (Lane et al., 2017b). Whether these patients may benefit also from silencing agents against SAA, which have been studied in the murine model, is presently unknown (Kluve-Beckerman et al., 2011).

**Figure 2:**
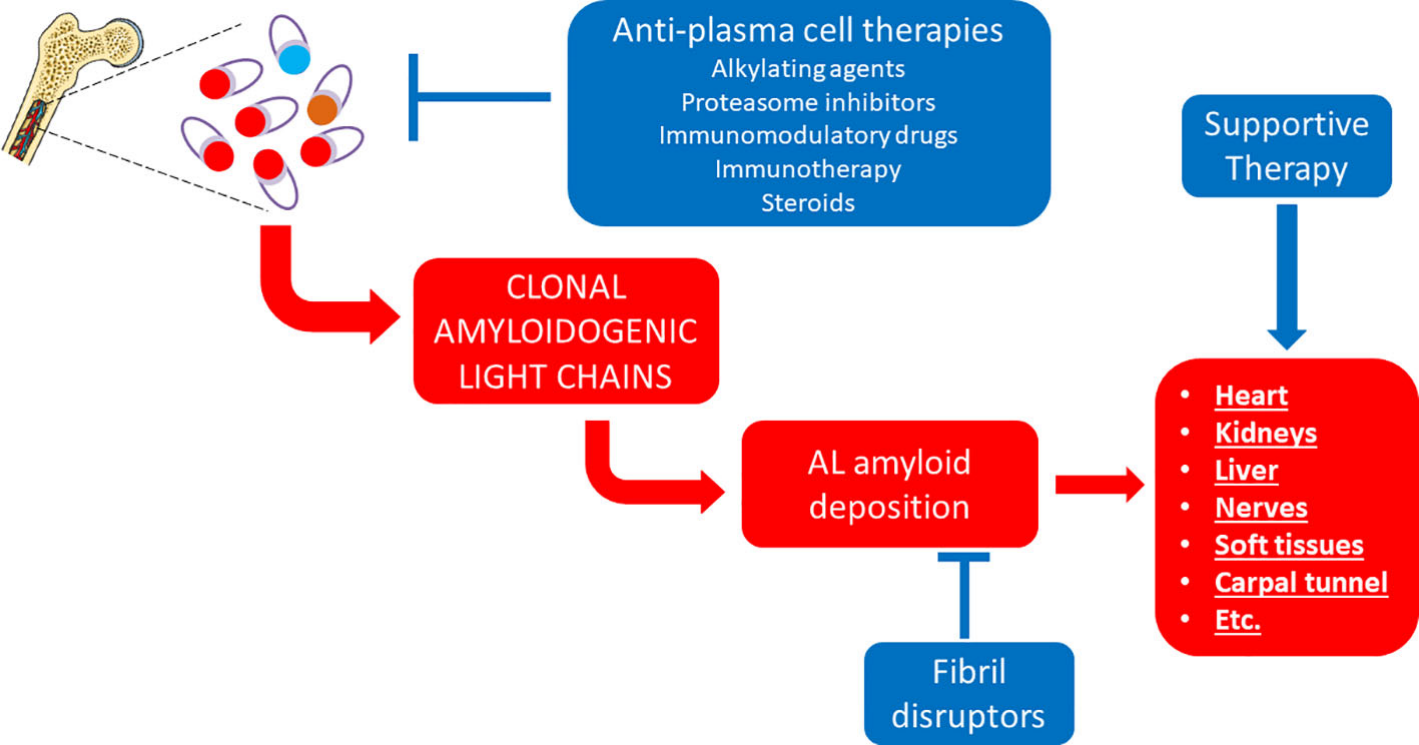
Established therapeutic interventions against AL amyloidosis. Red blocks indicate steps in the pathogenesis of AL amyloidosis; blue blocks indicate established therapeutic interventions against AL amyloidosis.

Even more satisfactory therapeutic results have been achieved in another form of systemic amyloidosis that is AL amyloidosis (**Figure 2**). This is caused by an underlying plasma cell (or B cell) clonal disorder resulting in the production of an unstable immunoglobulin light chain (**Table 4**), which can form amyloid in the heart, kidneys, liver, gastrointestinal tract, peripheral nerves, soft tissues and elsewhere, commonly in various combinations (Merlini et al., 2018). The obvious therapeutic strategy in this case has been to use drugs developed against other, more prevalent plasma cell (or B cell) disorders, including alkylating agents, steroids, proteasome inhibitors, immunomodulatory drugs and monoclonal antibodies (Merlini et al., 2018).

**Table 4:** Main clinical conditions associated with AL amyloidosis based on type of M protein and underlying clonal cell.

M protein type	Clonal cell	Related conditions
Non-IgM	Plasma cell	AL
		MM + AL
		PCL + AL
		B-cell CLL + AL
		B-cell NHL + AL
		Other
IgM	B cell (exceptionally plasma cell)	AL with IgMκ or IgMλ clone
		WM + AL
		B-cell CLL + AL
		B-cell NHL + AL
		(exceptionally IgM MM + AL)
		Other

AL, immunoglobulin light chain amyloidosis; CLL, chronic lymphocytic leukemia; MM, multiple myeloma; NHL, non-Hodgkin lymphoma; PCL, plasma cell leukemia; WM, Waldenström macroglobulinemia.

High dose melphalan followed by rescue therapy with autologous stem cell transplantation (ASCT) for the fittest, transplant-eligible subset of AL amyloidosis patients grants the most durable responses (Sanchorawala et al., 2015; Sidiqi et al., 2018; Sidana et al., 2019). Scrupulous selection of transplant eligible patients has dramatically reduced transplant-related mortality in more recent patients’ series (Sidiqi et al., 2018). When full dose melphalan (200 mg/m^2^) is employed, the median overall survival achieved is superior to 10 years (Sanchorawala et al., 2015; Sidiqi et al., 2018). In patients with bone marrow plasma cell infiltration above 10%, induction therapy before ASCT to expedite lowering of toxic light chains may improve rates of hematologic response and overall survival (Hwa et al., 2016; Scharman et al., 2017), stressing the importance of swiftly turning off the production of the amyloid-forming protein (Mikhael, 2018).

Transplant-ineligible patients due to advanced disease or significant comorbidity are treated with standard or attenuated doses of chemotherapy against the plasma cell clone, using alkylating agents, proteasome inhibitors, immunomodulatory drugs, monoclonal antibodies and steroids (Merlini et al., 2018). Several drug combinations are available and they are associated with median survivals exceeding 5 years, with the exception of patients with very advanced cardiac involvement at diagnosis, who show dismal prognosis irrespectively of the employed regimen (Merlini et al., 2018).

The extent of reduction of the amyloidogenic light chains with respect to pre-therapy levels can be used to grade the depth of hematologic response to therapy into four main response categories, that is no response, partial response, very good partial response and complete response, which are characterized by a progressively better prognosis (Palladini et al., 2012). Indeed, drop of the amyloidogenic, clonal light chain is paralleled by reduction of markers of heart dysfunction (Palladini et al., 2006) and hematologic response to therapy is the prerequisite to halt organ function deterioration or even promote organ function recovery (i.e. organ response to therapy) (Dispenzieri et al., 2006; Palladini et al., 2012; Palladini et al., 2014; Muchtar et al., 2018). Of note, the extent and rapidity of amyloidogenic light chain reduction is associated with higher rates of organ response and longer event-free survival (Palladini et al., 2020).

Among patients achieving the hematologic complete response status, that is the disappearance of M protein/monoclonal light chains and the normalization of serum free light chain levels, next generation flow cytometry or next generation sequencing can be employed to identify residual clonal plasma cells (defined as minimal residual disease, MRD) within the bone marrow with high (10^−5^–10^−6^) sensitivity (Kastritis et al., 2018). Recent studies are indicating that AL patients with MRD persistence have lower rates of organ response (Sidana et al., 2020; Staron et al., 2020). While the biologic bases of this observation are not clear, one possibility is that low levels of toxic, amyloidogenic light chains, below detection limits of routine diagnostic tests, may be enough to prevent organ function restoration. This highlights the importance of completely eradicating the underlying plasma cell clone, to arrest further damage and recover organ function.

**Figure 3:**
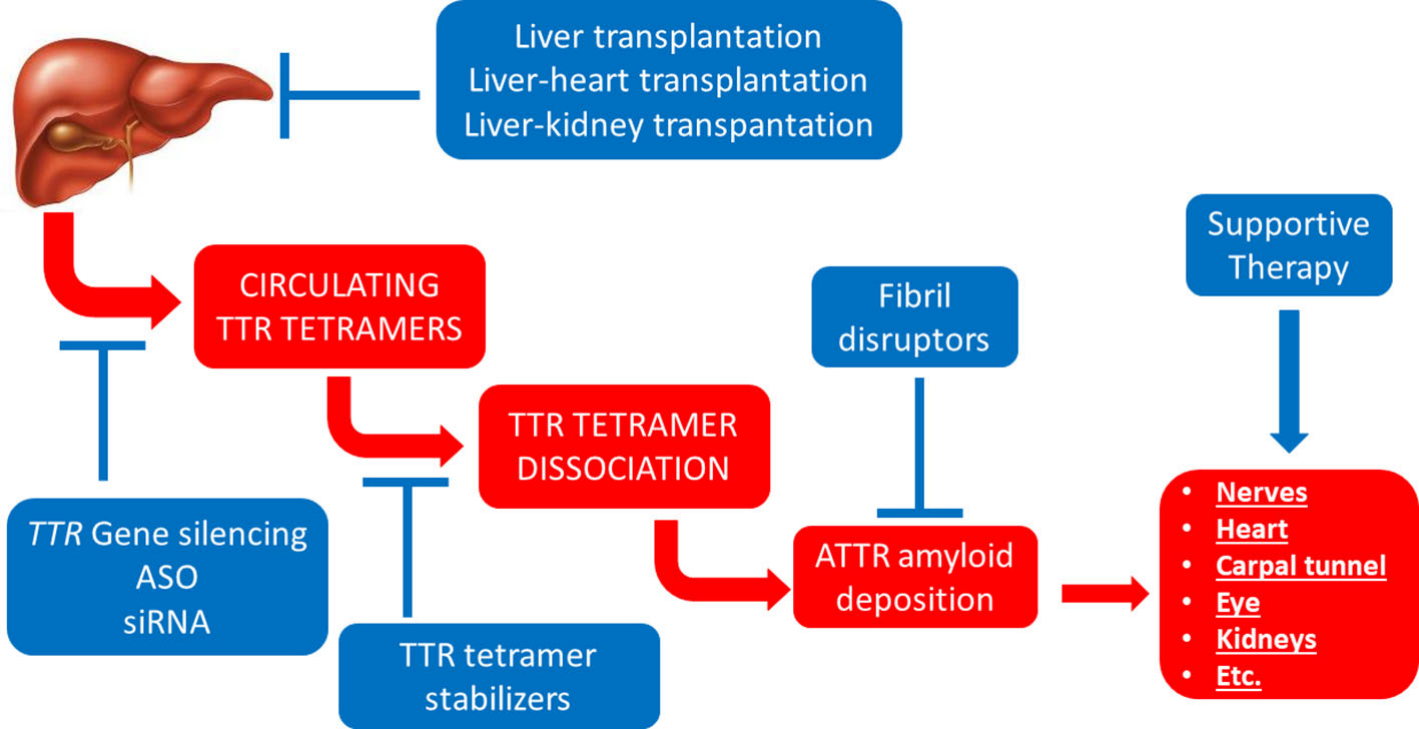
Established therapeutic interventions against ATTR amyloidosis. Red blocks indicate steps in the pathogenesis of ATTR amyloidosis; blue blocks indicate established therapeutic interventions against ATTR amyloidosis. ASO, anti-sense oligonucleotides; siRNA, short interfering RNA.

More recently, the elucidation of the molecular mechanisms underlying the pathogenesis of ATTR amyloidosis has enabled the development of novel targeted therapies against this disease (Ruberg et al., 2019) (**Figure 3**).

Circulating TTR is a homo-tetrameric protein mainly synthetized by the liver. Transthyretin is intrinsically amyloidogenic and can form amyloid also in its wild-type form, mainly within the heart, leading to ATTRwt amyloidosis (formerly senile systemic amyloidosis or senile cardiac amyloidosis) affecting predominantly elderly men. This is the result of a presumably very slow process which can become clinically manifest in elderly subjects or remain unappreciated and be detected as a *post-mortem* finding. Indeed, cardiac amyloid deposits containing TTR can be detected in 12–25% of subjects older than 80 years at autopsy (Cornwell et al., 1983; Tanskanen et al., 2008; Ueda et al., 2011). ATTRwt amyloidosis is an increasingly recognized cause of amyloid cardiomyopathy whose clinical detection has been conspicuously boosted by scintigraphy with bone tracers and increased awareness (Ravichandran et al., 2020). Of note, scintigraphy with bone tracers enabled to detect ATTRwt amyloidosis in 14–16% of patients with severe symptomatic aortic stenosis listed for transcatheter aortic valve replacement (Castano et al., 2017; Cavalcante et al., 2017; Scully et al., 2018; Scully et al., 2020).

Tetramer dissociation is currently regarded as the rate limiting factor for ATTR amyloidogenesis, as it releases TTR monomers which can then misfold and aggregate. A parallel mechano-enzymatic amyloidogenic mechanism has also been proposed (Marcoux et al., 2015). The presence of one of an ever-growing list of TTR tetramer-destabilizing mutations is the cause of hereditary or variant ATTR amyloidosis (ATTRv), which can affect peripheral nerves (hereditary ATTR amyloidosis with polyneuropathy, also known as familial amyloid polyneuropathy), the heart (hereditary ATTR amyloid cardiomyopathy, also known as familial amyloid cardiomyopathy) or both (at various degrees), based on the underlying *TTR* gene mutation (Connors et al., 2003; Rowczenio et al., 2019), with the neurotropic Val30Met (p.Val50Met) and the cardiotropic Val122Ile (p.Val142Ile) mutations being at the two extremities of the disease spectrum (Rapezzi et al., 2010). ATTRv amyloidosis associated to the Val30Met mutation mainly affects the peripheral and autonomous nervous systems (Andrade, 1952; Saraiva, 2002), but may involve the heart and the kidney. The disease is endemic in some areas of Portugal, Japan and Sweden, even though substantial differences in disease penetrance, age of onset (early or late onset) and other biochemical and clinical features exist among these geographic areas (Araki et al., 1968; Andersson, 1976; Araki, 1984; Alves et al., 1997). The Val122Ile *TTR* mutation is present in approximately 4% of African Americans and can lead to late-onset restrictive amyloid cardiomyopathy (Quarta et al., 2015).

For long time, liver transplantation to abolish variant TTR production as a sort of surgical gene therapy has been the only available therapeutic option in selected ATTRv patients. The best results are seen in patients with early-onset hereditary ATTR amyloidosis with polyneuropathy associated with the Val30Metmutation transplanted at early disease stages (Carvalho et al., 2015).

While most of the mutations in the *TTR* gene destabilize TTR tetramer and favor amyloid formation, the Thr119Met, or p.Thr139Met, mutation stood out for its unprecedented properties. Indeed, this mutation was identified in compound heterozygous subjects who were protected from the development of amyloid deposits despite the presence of a highly prevalent amyloidogenic mutation (the Val30Met mutation in the endemic Portuguese area) (Coelho et al., 1993). Subsequent biochemical and biophysical studies clarified that the protective effect of the Thr119Met mutation was due to the stabilization of TTR hetero-tetramer formed by both Val30Met and Thr119Met monomers (Hammarstrom et al., 2001). Collectively, these clinical and laboratory observations paved the way for a novel therapeutic approach against TTR-related amyloidosis, that is TTR tetramer stabilization (Hammarstrom et al., 2003). Both structure-based drug design and drug repurposing were pursued to identify TTR stabilizers with therapeutic effects.

The non-steroidal anti-inflammatory drug diflunisal was identified as a TTR stabilizer, with a >1 stoichiometry to serum TTR when orally administered thanks to its high bioavailability (Hammarstrom et al., 2003; Sekijima et al., 2006). An international randomized, double-blind, placebo-controlled study on patients with hereditary ATTR amyloidosis with polyneuropathy showed that diflunisal administration for 2 years reduced the rate of progression of neurological impairment and preserved quality of life (Berk et al., 2013).

The first drug to obtain approval for the treatment of ATTRv amyloidosis, tafamidis, was the result of structure-based drug design. This molecule was indeed designed to bind to one of the two T4-binding pockets of the TTR tetramer and to slow tetramer dissociation (Razavi et al., 2003; Bulawa et al., 2012). Its efficacy was tested in the context of a multicenter, international, randomized, placebo-controlled clinical trial. Daily treatment of patients affected by hereditary ATTR amyloidosis with polyneuropathy (*TTR* Val30Met mutation) with tafamidis led to circulating TTR tetramer stabilization and, in the efficacy-evaluable cohort of patients, to a significant delay in peripheral neurologic impairment over a 18-month treatment period compared to placebo, even though the coprimary endpoints of the trial were not met (Coelho et al., 2012). Subsequent case series confirmed tafamidis efficacy also in additional patients’ cohorts, including in patients with non-Val30Met*TTR* mutations and in patients with ATTRv cardiomyopathy (Coelho et al., 2013b; Merlini et al., 2013; Damy et al., 2015; Cortese et al., 2016; Ishii et al., 2019). Another clinical trial assessed tafamidis efficacy against ATTR cardiomyopathy (both ATTRv and ATTRwt types). Daily treatment with tafamidis was associated with reductions in all-cause mortality and cardiovascular-related hospitalizations, along with reduced decline in functional capacity and quality of life as compared with placebo (Maurer et al., 2018). The outcome of this trial led to the FDA and EMA approval of tafamidis as the first drug for the treatment of ATTR amyloidosis cardiomyopathy.

An additional TTR tetramer stabilizer developed based on structural data is represented by AG10. This small molecule stabilizes both wild-type and Val122Ile-containing TTR tetramers from patients’ sera (Penchala et al., 2013; Miller et al., 2018). Twice daily treatment of patients with ATTR cardiomyopathy (both ATTRv and ATTRwt) with AG10 led to a dose-dependent stabilization of TTR tetramers and to increased circulating TTR levels (Judge et al., 2019). An ongoing phase 3 trial is investigating the efficacy and safety of AG10 compared to placebo administered on a background of stable heart failure therapy in patients with ATTRv or ATTRwt cardiomyopathy (NCT03860935).

Other rationally designed small molecule TTR tetramer binders, including the palindromic molecule mds84, which simultaneously occupies both T4-binding pockets of TTR tetramers (Kolstoe et al., 2010; Corazza et al., 2019), are in preclinical development.

Screening of molecules under clinical development or already in use in humans led to the discovery that tolcapone, a drug used to treat patients with Parkinson’s disease, is a potent TTR tetramer binder, which can bind TTR in human plasma, stabilize TTR tetramers *in vivo* and inhibit TTR cytotoxicity (Sant’Anna et al., 2016; Gamez et al., 2019). As tolcapone is capable of passing the blood-brain-barrier, this molecule is particularly promising for the treatment of the clinical manifestations associated with the deposition of amyloid within the central nervous system, which are seen especially in the presence of selected *TTR* mutations as a result of local TTR production at the choroid plexus. A clinical trial assessing the TTR tetramer stabilizing effect of tolcapone in plasma and cerebrospinal fluid of patients with symptomatic ATTRv leptomeningeal amyloidosis and asymptomatic *TTR* mutation carriers has recently completed recruitment (NCT03591757).

More recently, an alternative therapeutic approach against TTR-related amyloidosis has been explored, one which is based on gene silencing. This has been achieved through either anti-sense oligonucleotides (ASOs) or silencing RNA (siRNA), which ultimately lower mature *TTR* mRNA levels within the liver, thus reducing hepatic TTR protein synthesis and secretion. As both wild-type and mutant TTR are found in the amyloid deposits, gene silencing agents have been designed to target highly conserved portions of the 3’-untranslated region of both wild-type and mutant *TTR* transcripts (Coelho et al., 2013a; Ackermann et al., 2016). Two large international randomized, double-blind, placebo-controlled studies demonstrated the efficacy of gene silencing agents for hereditary ATTR amyloidosis with polyneuropathy. Subcutaneous injection of the antisense oligonucleotide inotersen (three injections in the first week followed by weekly injections) over a 15-month period led to a median reduction in serum TTR concentration of 79% and improved the course of neurologic disease and health-related quality of life (Benson et al., 2018b). Intravenous administration of the siRNA agent patisiran once every 3 weeks over an 18-month period led to a median reduction in serum TTR concentration of 81% and improved several clinical manifestations of the disease compared to placebo (Adams et al., 2018). Remarkable findings were observed in the subset of enrolled patients displaying concomitant amyloid cardiomyopathy. In these patients, patisiran treatment was associated with echocardiographic features of better cardiac structure and function, reduced levels of the cardiac biomarker NT-proBNP and decreased adverse cardiac outcome, suggesting that this drug may halt or even reverse the progression of cardiac manifestations of hereditary ATTR amyloidosis (Adams et al., 2018; Minamisawa et al., 2019; Solomon et al., 2019).

As a result of the unprecedented successes seen in recent years, several alternative effective drugs are now available which can potentially reduce the availability of the amyloid-forming protein and interfere with the process of amyloid formation and organ damage for both AL and ATTR amyloidosis. Considering the paucity (or even the absence) of controlled clinical trials directly comparing different treatment regimens, now the question is how to select the best drug for each patient.

In AL amyloidosis, retrospective data from large series of homogenously treated patients have highlighted a potential role of molecular cytogenetics in predicting sensitivity of the underlying plasma cell clone to some anti-plasma cell drugs. Gain of chromosome 1q21 is an independent adverse prognostic factor in patients treated with melphalan and dexamethasone (Bochtler et al., 2014). Conversely, translocation t(11;14) is associated with adverse outcome in newly diagnosed and transplant-ineligible patients who are treated with bortezomib-based regimens (Bochtler et al., 2015). Interestingly, the survival disadvantage wrought by the presence of the t(11;14) translocation can be abrogated by exposure to melphalan (Muchtar et al., 2017). Based on these observations, the combination of bortezomib, melphalan and dexamethasone, which has been compared to treatment with melphalan and dexamethasone alone in the context of a clinical trial (NCT01277016), has the potential to by-pass the reduced sensitivity of gain 1q21- or t(11;14)-positive plasma cell clones towards melphalan or bortezomib, respectively.

The t(11;14) translocation, which is present in up to 60% of AL plasma cell clones, results in the overexpression of the anti-apoptotic BCL-2 protein. The selective BCL-2 inhibitor venetoclax showed therapeutic effect in a few cases of relapsed/refractory AL amyloidosis (Leung et al., 2018; Ghilardi et al., 2019; Premkumar et al., 2019), in line with positive findings against relapsed/refractory t(11;14)-positive multiple myeloma cases, both as a single agent or in combination with bortezomib (Kumar et al., 2017; Moreau et al., 2017).

In ATTR amyloidosis, retrospective data from a large series of patients with hereditary ATTR amyloidosis and polyneuropathy treated with tafamidis were analyzed to identify possible predictors of tafamidis response. This analysis enabled the definition of a prognostic model based on patient’s sex, disease severity and native TTR concentration at the outset of treatment to predict response to this TTR tetramer stabilizer (Monteiro et al., 2019).

## Monitoring the Concentration of the Amyloid-Forming Protein Guides Therapeutic Interventions

Experimental and clinical evidence point towards a crucial pathogenetic role of soluble oligomeric intermediates of fibril assembly as the main mediators of cytotoxicity and organ damage (Comenzo et al., 1996; Lambert et al., 1998; Hartley et al., 1999; Dember et al., 2001; Sousa et al., 2001; Andersson et al., 2002; Walsh et al., 2002). However, such oligomeric species remain difficult to be measured through routine methods. In the impossibility of directly assessing levels of oligomeric species, the effect of etiologic interventions against AA, AL and ATTR amyloidosis can be assessed by closely monitoring circulating levels of the amyloid-forming protein (that is, SAA, circulating free light chains, and TTR, respectively).

In AA amyloidosis, SAA levels above 10 mg/L should justify a potentiation of the anti-inflammatory therapy, as levels above this threshold value are associated to a significantly increased risk of organ damage progression or amyloid recurrence in renal graft for transplanted patients (Pinney et al., 2013).

In AL amyloidosis, the amount of the amyloid-forming protein, as indicated by baseline immunoglobulin free light chain (FLC) levels, bears a negative prognostic effect (Dispenzieri et al., 2006). Also, the difference between involved and uninvolved FLC (denoted as dFLC, which is indicative of the burden of amyloidogenic light chains in patients with AL amyloidosis) is a negative risk factor independent of the extent of cardiac involvement (Kumar et al., 2012). In this context, the aim of anti-plasma cell therapy is to profoundly reduce or possibly eliminate the amyloidogenic light chain. Reaching adFLC of <40 mg/L or less was considered the aim of therapy (Palladini et al., 2012). However, recent evidence has emerged indicating that a concentration of dFLC of 10 mg/L or less after therapy (termed stringent dFLC response) is associated with improved outcome (Manwani et al., 2019). Also in patients with low levels of dFLC at diagnosis, who have less frequent and less severe heart involvement and a better prognosis, achievement of dFLC below 10 mg/L after therapy is associated with a better overall survival and organ response (Dittrich et al., 2017; Milani et al., 2017; Sidana et al., 2018).

As for TTR, it should be noted that tetramer stabilizers and gene silencers lead to opposing changes in serum TTR levels, with the former leading to increased levels due to tetramer stabilization and the latter leading to reduced levels due to suppressed synthesis. Of interest, in the clinical trial investigating the therapeutic effect of patisiran in patients with hereditary ATTR amyloidosis with polyneuropathy, a correlation between the degree of the reduction in TTR levels from baseline and neurological manifestations was observed (Adams et al., 2018). In line, analysis of serum TTR levels from a large series of patients after 12 months of treatment with tafamidis showed higher levels of circulating TTR in patients classified as responders to tafamidis, compared to patients defined as partial responders or non-responders based on clinical evaluations (Monteiro et al., 2019).

For those cases in which experimental therapeutic approaches against the amyloid-forming protein are planned, the availability of reliable diagnostic assays to accurately monitor the levels if the amyloid-forming protein in the relevant compartment represents an essential prerequisite for clinical trial design and patient management. In the case of prion diseases, where neurodegeneration is due to replication of the endogenous prion protein in the presence of the infectious agents, the prion, there are efforts at developing silencing agents to lower *PRNP* expression and reduce cellular prion protein expression, with the hope to attenuate prion-induced neurodegeneration or even prevent it in at risk individuals (e.g. pre-symptomatic carriers of highly penetrant mutations) (Nuvolone et al., 2009). The validity of this approach is corroborated by observations in animal models (Mallucci et al., 2003; Mallucci et al., 2007). In this context, the development of a robust diagnostic assay to measure prion protein levels in cerebrospinal fluids (Vallabh et al., 2019), together with ongoing animal studies on the therapeutic effect of *PRNP* silencing agents, are welcomed achievements in the pathway leading to clinical trials against these as of yet orphan diseases.

## Reduction/Elimination of the Amyloid-Forming Protein may Delay/Prevent Disease Onset

Whenever possible, prevention can be highly effective and can significantly impact on the epidemiology of the disease and modify incidence and/or prevalence. This has been achieved so far—even though only for a few forms of systemic amyloidosis—by avoiding increased concentrations of the amyloid-forming protein in at risk individuals.

**Figure 4:**
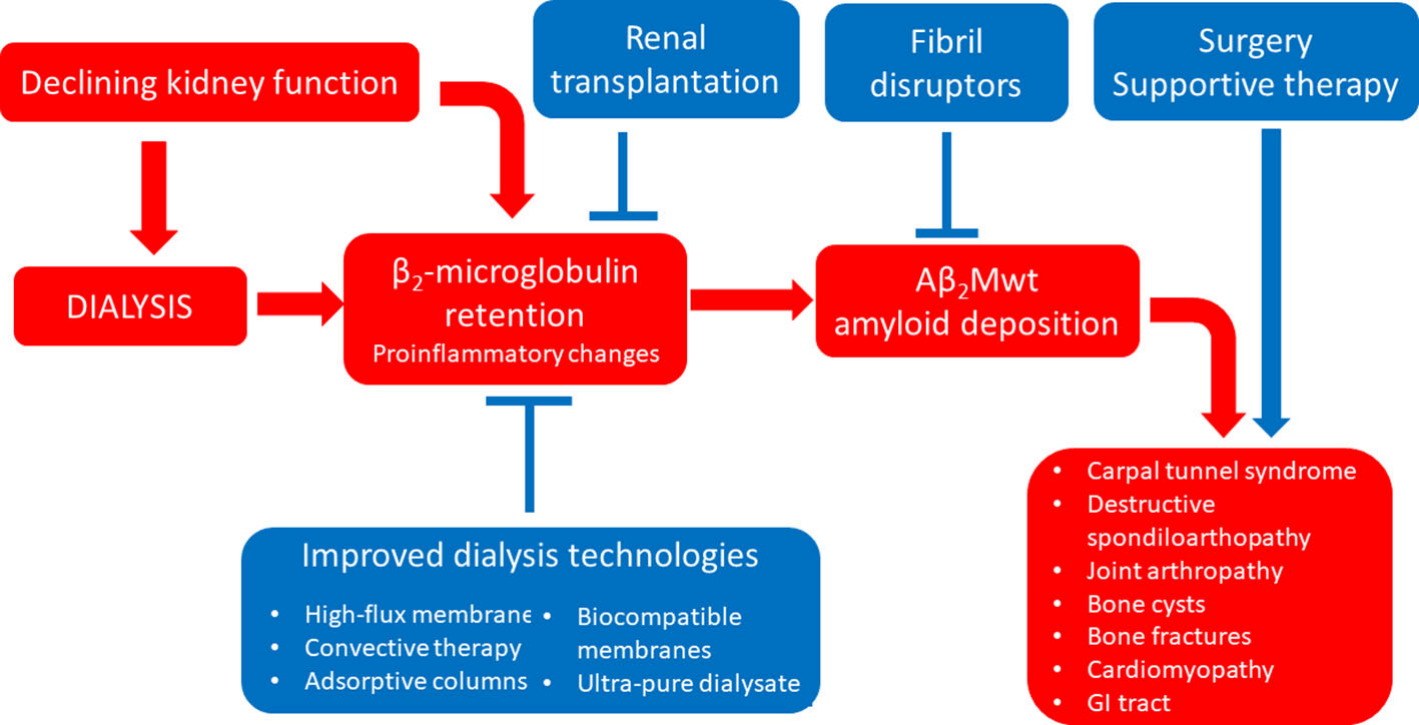
Established therapeutic interventions against Aβ_2_Mwt amyloidosis. Red blocks indicate steps in the pathogenesis of Aβ_2_Mwt amyloidosis; blue blocks indicate established therapeutic interventions against Aβ_2_Mwt amyloidosis. GI tract, gastro-intestinal tract.

Amyloidosis associated with wild-type β_2_-microglobulin (Aβ_2_Mwt amyloidosis, also known as dialysis-related amyloidosis) can occur in patients undergoing long-term hemodialysis (occasionally also in patients in peritoneal dialysis or in patients with end stage renal disease not yet on dialysis) (Zingraff et al., 1990). Because of lack of efficient renal catabolism and clearance of β_2_-microglobulin, circulating levels of this protein are chronically increased in these patients, and this can result in the formation of amyloid deposits, most commonly within the osteo-articular system (Scarpioni et al., 2016). *Post-mortem* studies showed that this condition can be detected histologically in almost 50% of hemodialysis patients at autopsy, even though the disease becomes clinically or radiologically evident only in a subset of these cases (Jadoul et al., 1997). Over the last two decades, significant advances in dialysis technology, including the use of high flux membranes, convective regimens, adsorptive columns/membranes and biocompatible materials have succeeded at reducing β_2_-microglobulin levels in dialysis patients (**Figure 4**). In line, these improvements have resulted in a significant delay of the occurrence of Aβ_2_Mwt amyloidosis, leading to overall reduced prevalence and severity of this invalidating complication of long-term dialysis (Schiffl, 2014; Hoshino et al., 2016; Morris et al., 2019).

In the case of AA amyloidosis, a better control of the inflammatory status through more effective drugs, including disease-modifying anti-rheumatic drugs and monoclonal antibodies, is the most plausible explanation for the reduced incidence of this type of amyloidosis in high income Countries (Lane et al., 2017a).

In principle, this approach could be extended to other protein misfolding and deposition diseases. A promising clinical setting for preemptive interventions is represented by asymptomatic carriers of amyloidogenic *TTR* mutations, especially in the era of effective silencing agents. There are currently no data to support the potential benefit of preemptive interventions in pre-symptomatic mutation carriers. Because of the existence of incomplete penetrance, the first setting where to test this approach may be *TTR* Val30Met carriers from endemic areas with high disease penetrance. In this context, the elucidation of the bases of susceptibility, including the study of potential modifier genes (Santos et al., 2016; Santos et al., 2018; Dias et al., 2019; Santos et al., 2019), along with the development of high-sensitivity imaging modalities to detect minute amount of amyloid deposits at the pre-symptomatic stage (Kollmer et al., 2015; Kollmer et al., 2017) would help to identify individuals who could benefit most from such a preemptive intervention.

For ATTRwt amyloidosis, no known predisposing factors have been identified so far beyond aging and male sex, even though an association with hip and knee arthroplasty, carpal tunnel syndrome, spinal canal stenosis and aortic stenosis has been described (Rubin et al., 2017; Aus dem Siepen et al., 2019; Scully et al., 2020).

In AL amyloidosis, at risk individuals are represented by subjects with a known monoclonal gammopathy. However, only a small proportion of patients with MGUS, MM or other plasma cell dyscrasia eventually develop AL amyloidosis.

There are current research efforts aiming at identifying individuals at higher risk of developing AL amyloidosis among subjects with monoclonal gammopathy. One such effort exploits the known association of AL amyloidosis with a few germ-line genes encoding for the variable light chain gene, VL, possibly due to the high amyloidogenicity of these germ-line genes. In particular, patients with a monoclonal protein expressing the *IGLV6-57*, *IGLV1-44*, *IGLV2-14* or *IGLV1-51* have a relative risk of developing AL amyloidosis vs myeloma ranging from 7.3, 2.5, 1.6 to 1.2, respectively (Zhou et al., 2019) (NCT02741999). Based on this, the impact of ascertaining the VL gene family through sequencing approaches in bone marrow specimens in patients with MGUS/monoclonal gammopathy is currently being explored (Zhou et al., 2019).

Another approach is based on the functional characterization of patient-derived monoclonal light chains. Indeed, a functional competition assay based on *in vitro* fibrillization has been established. The test succeeded at differentiating amyloidogenic from control light chains, based on the superior capacity of the formers to inhibit *in vitro* recruitment of an amyloidogenic variable light chain domain by homologous amyloid-like fibrils (Martin et al., 2017; Martin et al., 2018).

Recent interesting data have emerged from the study of post-translational modifications of monoclonal light chains from the serum of patients with AL amyloidosis or other plasma cell disorders. In particular, the use of mass spectrometry on immunopurified light chains from patients’ serum has identified distinctive M protein mass distributions patterns possibly indicating N-glycosylation of monoclonal light chains in 33 and 10.2% of patients with AL-κ and AL-λ amyloidosis, compared to 3.7 and 4.9% in patients with non-AL monoclonal gammopathies of κ and λ type, respectively (Kumar et al., 2019). Subsequent analyses in a subset of cases involving enzymatic deglycosylation and mass spectrometric analysis confirmed the presence of N-glycosylation in the analysed monoclonal light chains (Kumar et al., 2019). These observations are in line with previous studies on a limited number of amyloidogenic light chains showing N-glycosylation as a post-translational modification detected in a subset of cases (Stevens, 2000). As mass spectrometry analysis is an emerging area for the screening of M proteins, the identification of potential N-glycosylation in monoclonal light chain may help identifying subjects at higher risk of developing AL amyloidosis (Kumar et al., 2019).

Additional studies are needed to show the potential clinical application of these different approaches and their impact on patients’ outcome.

## Early Diagnosis is Vital

As systemic amyloidoses are characterized by the progressive organ dysfunction of affected organs, early diagnosis is of utmost importance in order to intercept patients before the development of irreversible organ damage.

A delayed diagnosis, in the presence of advanced organ damage and possibly of multi-systemic involvement, unavoidably results in increased frailty, bears negative prognostic impact and represents a significant limitation for therapy.

In the general population of patients with AA amyloidosis, more than 10% showed end-stage renal disease at diagnosis (Lachmann et al., 2007; Palladini et al., 2017b). This figure increases up to ≈25% in patients developing AA amyloidosis as a complication of hereditary autoinflammatory syndromes (Lane et al., 2013). Proteinuria-based screening should be considered to promote early recognition of AA amyloidosis in at risk-patients with chronic inflammatory diseases (Papa and Lachmann, 2018).

In a series of 108 patients with ATTRwt amyloidosis, approximately one third of subjects had severe heart failure (New York Heart Association, NYHA, class III or IV) at diagnosis (Gonzalez-Lopez et al., 2017). Of note, in the clinical trial testing the therapeutic effect of tafamidis-mediated TTR tetramer stabilization in patients with ATTR amyloid cardiomyopathy, tafamidis reduced the rate of cardiovascular-related hospitalization in all patients’ subgroups, except patients with NYHA class III (patients with NYHA IV class were excluded from the trial). This again stresses the importance of an earlier diagnosis and a prompt initiation of therapy. Luckily, the increased awareness of this disease, along with the increased role of cardiac imaging and the possibility of a biopsy-free diagnosis through scintigraphy with radiotracers (Gillmore et al., 2016) facilitate the diagnosis of ATTR amyloid cardiomyopathy at earlier disease stages. Indeed, among ATTRwt amyloidosis patients evaluated at a single institution, patients diagnosed after the introduction of a scintigraphy-based non-invasive diagnosis showed lower rate of advanced disease stage compared to patients diagnosed earlier (Lane et al., 2019). In line, recent real-world case series of newly diagnosed ATTRwt amyloidosis patients show milder disease stages compared to cohorts of patients enrolled in clinical trials in terms of NYHA functional class, hemodynamic parameters and cardiac biomarkers (Canepa et al., 2019).

As carpal tunnel syndrome, often bilateral, is present in 20–50% of ATTRwt amyloidosis patients at diagnosis (Nakagawa et al., 2016; Gonzalez-Lopez et al., 2017; Milandri et al., 2020) as well as in 10–20% of AL amyloidosis patients (Prokaeva et al., 2007; Rapezzi et al., 2009), and in most cases this condition precedes cardiac (or other) manifestations of the diseases by many years, different screening approaches have been applied in this clinical setting. Congo red staining of tenosynovial tissue has been employed to detect amyloid deposits at this site in patients with carpal tunnel syndrome (males aged ≥50 years and females ≥60 years) undergoing carpal tunnel release surgery. Amyloid deposits were found in 10% of evaluated patients, enabling the diagnosis of previously unrecognized amyloidosis (ATTRwt, ATTRv and AL), including symptomatic patients requiring therapy (Sperry et al., 2018). In another study, patients aged 60 years or older who underwent carpal tunnel release surgery were evaluated by echocardiography and, in the presence of increased left ventricular hypertrophy, by additional tests to ascertain the presence of cardiac amyloidosis. Cardiac amyloidosis (ATTRwt and AL) was identified in 1% of screened patients and this figure increased to >10% when focusing on patients with bilateral carpal tunnel syndrome and left ventricular hypertrophy (Zegri-Reiriz et al., 2019).

Also in the case of hereditary ATTR amyloidosis with polyneuropathy, administration of tafamidis at earlier disease stages led to a greater preservation of neurologic function compared to patients in which tafamidis was initiated later (Coelho et al., 2013b; Plante-Bordeneuve et al., 2017).

In AL amyloidosis, a delayed diagnosis is the rule rather than the exception. According to a recent patient-initiated survey, patients received a diagnosis after an average of 3 years from clinical onset and after consulting ≥4 specialists (McCausland et al., 2018). Not surprisingly, about 85% of AL patients are not eligible for ASCT at diagnosis due to advanced organ involvement. Noteworthy, a subset of these patients may eventually become transplant-eligible and undergo successful deferred ASCT upon response to standard dose, induction chemotherapy, as a result of reversal of organ dysfunction present at diagnosis, with excellent outcome (Manwani et al., 2018). Fifteen to 20% of AL amyloidosis patients show advanced cardiac involvement at diagnosis. These patients are particularly frail, have limited therapeutic options and have a dismal prognosis (Wechalekar et al., 2013).

Recently, a strategy to promote early diagnosis of AL amyloidosis, before overt symptomatic organ damage has been promoted. At diagnosis, virtually all (95%) AL amyloidosis patients have an abnormal serum immunoglobulin free light chain ratio and >80% of cases have increased NT-proBNP levels and/or proteinuria due to heart and/or kidney involvement. Based on these figures, the addition of NT-proBNP and proteinuria within the battery of biomarkers employed for the regular follow up of patients with MGUS has been advocated for those MGUS cases with an abnormal free light chain ratio (Merlini and Palladini, 2012). Such a strategy has indeed succeeded at identifying pre-symptomatic patients with AL amyloidosis, at early disease stages, resulting in excellent outcomes (Palladini et al., 2017a). As AL amyloidosis is often diagnosed late also in patients with a known monoclonal gammopathy under hematologic follow up (Kourelis et al., 2014), this approach has the potential to significantly reduce the diagnostic delay. In general, increasing the awareness of AL amyloidosis among physicians may reduce the patients’ journey before achieving a diagnosis (McCausland et al., 2018).

Prompt reduction of the amyloid-forming protein is necessary but not sufficient to guarantee restoration of organ function, improvement of quality of life and prolonged survival. Organ function recovery may depend on the type and severity of organ damage and possibly, also on the type of amyloid and on as of yet poorly identified inter-individual factors.

Lack of organ function restoration may be explained, at least in part, by the persistence of amyloid deposits within affected organs. Of note, among AA amyloidosis patients where anti-inflammatory therapies successfully lowered circulating SAA levels below 10 ng/L, amyloid regression was seen in 60% of cases and survival among these patients was superior to survival among those in whom amyloid deposits did not regress (Lachmann et al., 2007).

In this context, therapeutics able to promote amyloid clearance may facilitate restoration of organ function. This approach has attracted much attention in recent years, with three monoclonal antibodies targeting amyloid deposits being tested in clinical trials, and more molecules in the pipeline (Nuvolone and Merlini, 2017a). Despite promising preliminary results of NEOD001, a monoclonal antibody targeting misfolded amyloid proteins (Gertz et al., 2016), and of miridesap, a monoclonal antibody targeting amyloid-bound SAP after pharmacological depletion of circulating SAP (Richards et al., 2015; Richards et al., 2018), several clinical trials investigating these biologicals were recently interrupted due to futility analysis (for NEOD001, NCT02312206; NCT03168906) or to a change in benefit/risk profile (for miridesap, NCT03044353). A third chimeric fibril reactive monoclonal antibody, CAEL-101, formerly 11-1F4, is currently under clinical evaluation (Edwards et al., 2019) (NCT02245867).

An alternative strategy to promote amyloid clearance is based on the amyloid fibril disrupting activity of 4ʹ-iodo-4ʹ-deoxydoxorubicin and of the structurally related molecule doxycycline (Merlini et al., 1995; Cardoso et al., 2010). Several clinical observations support a potential benefit of doxycycline treatment against ATTR, AL and Aβ_2_Mwt amyloidosis (Obici et al., 2012; Montagna et al., 2013; Piccoli et al., 2017; Wechalekar and Whelan, 2017; Karlstedt et al., 2019) and several ongoing clinical trials are currently investigating the potential therapeutic effect of doxycycline against different forms of systemic amyloidosis.

## Conclusive Remarks

Recent, unprecedented progress in the treatment of different forms of systemic amyloidoses is rapidly changing the natural history of these diseases, significantly prolonging patients’ survival and even leading to effective preemptive interventions which result in reduced disease incidence in some instances. Still, systemic amyloidoses remain serious illnesses, which may lead to deterioration of organ function, poor quality of life and reduced survival, and only subsets of patients benefit from currently available therapies. Thus, there is still much space for improvements in the treatment of these diseases.

Clinical observations stemming from the therapeutic success achieved in the more common forms of systemic amyloidoses all converge at indicating the early and conspicuous reduction of the amyloid-forming protein as the presently most effective treatment strategy. Such observations also stress the importance of identifying these diseases early, to enable timely therapeutic interventions, to prevent advanced organ damage and to increase the likelihood of organ function restoration and improvement in quality of life and disease-free survival.

While the few tested interventions aimed at promoting amyloid clearance did not show clear clinical benefit against systemic amyloidoses so far, this therapeutic approach should be further pursued, as it could represent an important complementation to halt or reverse organ dysfunction.

Most of the success obtained in the treatment of AL amyloidosis is the result of the impressive progress made in recent years by academic and industrial research at developing drugs against the more prevalent plasma cell tumor multiple myeloma. This has enabled the use of novel, potent anti-plasma cell drugs to treat also patients with AL amyloidosis. Similarly, the changing paradigm seen in the context of AA amyloidosis, with reduced incidence of this type of amyloidosis and a relative increase of cases in which the underlying inflammatory condition cannot be unambiguously identified, reflects recent improvements in the treatment of inflammatory arthritides, chronic inflammatory bowel diseases and infections. While one could argue that most of the progress made in the treatment of AL and AA amyloidosis was serendipitous and just reflects improvements in the treatment of myeloma and chronic inflammatory conditions, on a more positive note the scenario is totally different in the case of TTR-related amyloidosis. Here, relentless, dedicated efforts at uncovering the molecular mechanisms of this disease and at identifying or designing novel effective drugs have eventually succeeded at providing tailored, targeted therapies against this disorder. This unprecedented therapeutic success was the result of the use of different approaches for drug discovery, including high-throughput screens, rational structure-based drug design and gene silencing. The common denominator of these approaches is to reduce the supply or to stabilize transthyretin, again stressing the importance of rapidly and profoundly turning off the supply of the amyloid-forming protein to treat amyloid diseases.

There is hope that the example of ATTR amyloidosis will not remain isolated, and that this will be replicated for the many forms of amyloidosis, either systemic or localized, for which etiologic therapies are still eagerly awaited.

## Author Contributions

AN, GM, and MN wrote and revised the manuscript. All authors contributed to the article and approved the submitted version.

## Funding

This work was supported by grants from the Italian Ministry of Health (Ricerca Finalizzata, grants RF-2013-02355259 and GR-2018-12368387), the CARIPLO Foundation (grants 2013-0964 and 2018-0257), the Amyloidosis Foundation, the “Associazione Italiana per la Ricerca sul Cancro—Special Program Molecular Clinical Oncology 5 per mille” (grant 9965) and by an Accelerator Award from the Cancer Research UK, the Fundación Científica—Asociación Española Contra el Cáncer and the Associazione Italiana Ricerca sul Cancro.

## Conflict of Interest

GM: consultant for Millennium Pharmaceuticals, Inc., Pfizer, Janssen, Prothena, and IONIS. MN: speaker honoraria from Janssen-Cilag.

The remaining author declares that the research was conducted in the absence of any commercial or financial relationships that could be construed as a potential conflict of interest.
